# Surgical management of hilar cholangiocarcinoma at Memorial Sloan Kettering Cancer Center

**DOI:** 10.1002/ags3.12181

**Published:** 2018-06-29

**Authors:** Michael E. Lidsky, William R. Jarnagin

**Affiliations:** ^1^ Department of Surgery Memorial Sloan Kettering Cancer Center New York NY USA

**Keywords:** complete surgical resection, hilar cholangiocarcinoma, portal vein embolization, preoperative biliary drainage

## Abstract

Hilar cholangiocarcinoma, which represents approximately 60% of biliary tract malignancies, is increasing in incidence and presents an ongoing challenge for patients and hepatobiliary surgeons. Although the majority of patients present with advanced disease, the remaining minority of patients are best treated with surgical resection or transplant. Transplant is typically reserved for locally unresectable tumors often in the setting of underlying hepatic dysfunction and will not be discussed herein. This review, therefore, focuses on oncological resection and the strategies implemented for the treatment of hilar cholangiocarcinoma at a quaternary referral center, including preoperative considerations such as patient selection and optimization of the future liver remnant, nuances to the operative approach for these tumors such as resection under low central venous pressure and management of the bile duct, as well as postoperative management.

## 1 INTRODUCTION

Hilar cholangiocarcinoma, first described by Altemeier in 1957,^1^ was popularized by Klatskin in 1965, who reported a series of patients presenting with jaundice, acholic stool, dark urine, and pruritis.^2^ In that era, a malignant process was suspected based on associated symptoms of anorexia, fatigue, and profound weight loss.^2^ The perioperative mortality rate in Klatskin's series was 92%, with 12 of 13 patients dying of hepatic failure or liver‐related complications.^2^ Fifty years later, hilar cholangiocarcinoma continues to challenge hepatobiliary surgeons, although significant improvements have been made. Indeed, the advent of more sophisticated imaging techniques has improved assessment of disease extent and patient selection for surgery, and preoperative interventions such as biliary drainage and portal vein embolization (PVE) have helped facilitate safer resection, which now has a perioperative mortality rate of 8% or less.^3^, ^4^

In the USA, cholangiocarcinoma occurs at a rate of two to three per 100 000 people, making it a rare disease. Estimates would suggest that the large majority arise in the extrahepatic biliary tree, with approximately 60% at the biliary confluence. Recently published data suggest that the incidence and mortality of biliary cancer generally, and extrahepatic tumors (hilar and distal) specifically, are both increasing.^5^

Unfortunately, the majority present with locally advanced or metastatic disease.^6^, ^7^ For the minority of patients with local disease only, resection and transplantation represent the only opportunities for cure. Retrospective data suggest that survival is similar after transplant and resection, after adjusting for age, tumor size, and nodal status.^8^ Given the obvious organ shortage, patients with resectable hilar cholangiocarcinoma should be treated with an R0 resection, reserving transplantation for patients with locally advanced disease that is beyond resectability and/or those with compromised hepatic function.^8^

**Figure 1:**
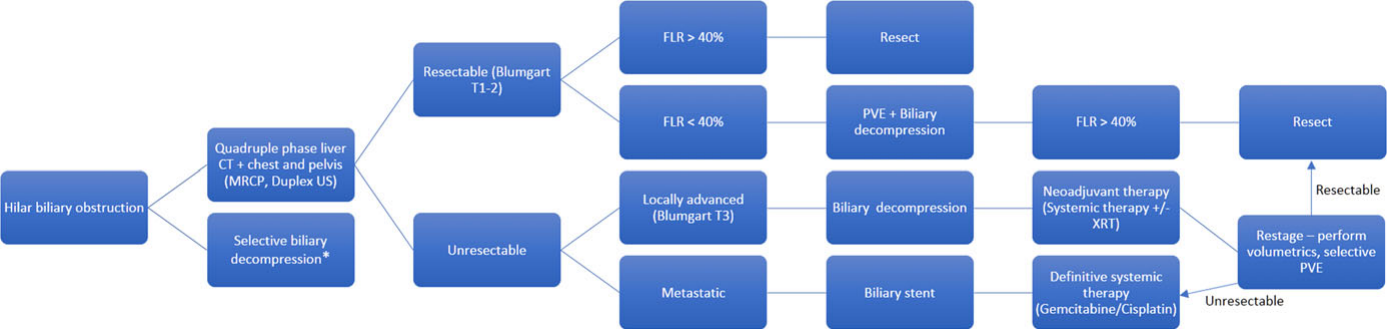
Algorithm illustrating the general approach to patients with hilar cholangiocarcinoma. *Patients presenting with obstructive cholangitis must undergo biliary decompression. FLR, future liver remnant; PVE, portal vein embolization

Surgical resection for hilar cholangiocarcinoma, at our institution and others, results in a 5‐year disease‐specific survival of approximately 40% with a median disease‐specific survival exceeding 40 months.^9^ Long‐term survival is limited by locoregional recurrence in 26% and distant metastasis in 40%, predicted by nodal and margin status as well as tumor differentiation, all of which are included in a predictive model of 3‐ and 5‐year disease‐specific survival.^9^, ^10^ This review focuses on oncological resection and the strategies implemented for the treatment of hilar cholangiocarcinoma at a quaternary referral center (Figure 1), including preoperative considerations such as patient selection and optimization of the future liver remnant (FLR), nuances to the operative approach for these tumors such as resection under low central venous pressure and management of the bile duct, as well as postoperative management. Key principles summarized herein are based on a thorough understanding of the available literature, which will be discussed when appropriate, but also largely on our institutional experience in caring for this rare and challenging malignancy.

## 2 PREOPERATIVE CONSIDERATIONS

### 2.1 Determining resectability

Diagnostic direct cholangiography, either endoscopic (endoscopic retrograde cholangiopancreatography, ERCP) or transhepatic (percutaneous transhepatic cholangiography, PTC), is rarely indicated in the era of modern imaging. Cross‐sectional imaging, therefore, is the cornerstone of disease assessment to identify patients with potentially resectable disease. Patients with intrahepatic metastases or distant disease do not benefit from resection and should be referred for palliative systemic therapy.^11^ For patients with localized disease, quadruple‐phase computed tomography (CT) or contrast‐enhanced magnetic resonance imaging (MRI with MRCP) are the imaging modalities of choice to determine whether resection is technically feasible.^12^ Ideally, these studies are obtained prior to biliary stenting, and then repeated after decompression of the biliary tree. Although the hilar mass may be difficult to visualize, high‐quality cross‐sectional imaging does provide essential information about proximity to or invasion of the biliary confluence, portal vein, and hepatic artery. For example, with tumors arising primarily from the left hepatic duct, the right hepatic artery may be involved or encased as a result of its posterior course in relationship to the bile duct, which is often a limiting factor for left parenchymal resection. Portal vein involvement is suggested on cross‐sectional imaging by narrowing or distortion of its normal contour, or obstruction of flow with resultant ipsilateral atrophy and contralateral lobar hypertrophy. Biliary obstruction causes intrahepatic biliary dilatation and similarly causes atrophy and hypertrophy of the ipsilateral and contralateral lobes of the liver, respectively. Additional information obtained from cross‐sectional imaging includes an assessment of portal lymph nodes. Metastatic disease to regional lymph nodes, if present, clearly has an adverse impact on survival and may influence treatment decisions. Although radiographically suspicious nodes may portend a poor prognosis, adenopathy may be reactive in the setting of biliary instrumentation, for example. Therefore, adenopathy on preoperative imaging should not necessarily preclude surgical exploration.

Two classification systems provide information about the primary lesion and its resectability. The Bismuth‐Corlette system relies on the extent of biliary involvement to stratify patients, without consideration of vascular invasion or resultant lobar atrophy. The staging system devised by Blumgart and colleagues at MSKCC does account for these variables and is therefore a more comprehensive preoperative tool to aid in determining resectability.^13^ The Blumgart T‐staging system was introduced in a 1998 publication that retrospectively analyzed 90 patients with hilar cholangiocarcinoma at MSKCC.^13^ Of the 69 patients that were explored, 39 (57%) were unresectable as a result of bulky adenopathy or distant metastases, whereas 30 (43%) were resected with an 83% negative margin rate.^13^ The proposed preoperative T‐staging system has since been modified and stratifies patients into three groups, according to the following factors: (i) tumor involvement of the biliary confluence versus bilateral hepatic ducts (Bismuth‐Corlette system); (ii) tumor invasion of the ipsilateral or contralateral portal vein; and (iii) the presence of lobar atrophy.^7^ Tumors classified as T1 were resectable in 59%, T2 in 31%, and T3 in 0%.^7^ Jarnagin et al^7^ and Matsuo et al^14^ reported that the preoperative T‐staging system correlated with resectability, likelihood of achieving an R0 resection, presence of metastatic disease, and median survival. Based on these data, the Blumgart preoperative T‐staging system, which includes radial and longitudinal tumor extension and involvement of adjacent biliary and vascular structures, has proven useful for patient selection for curative intent resection^7^, ^13^, ^14^ and also for selection of patients for staging laparoscopy, given the higher yield in patients with higher T‐stage tumors.^4^, ^15^

### 2.2 Volumetric analysis to identify patients at risk for postoperative hepatic insufficiency

**Figure 2:**
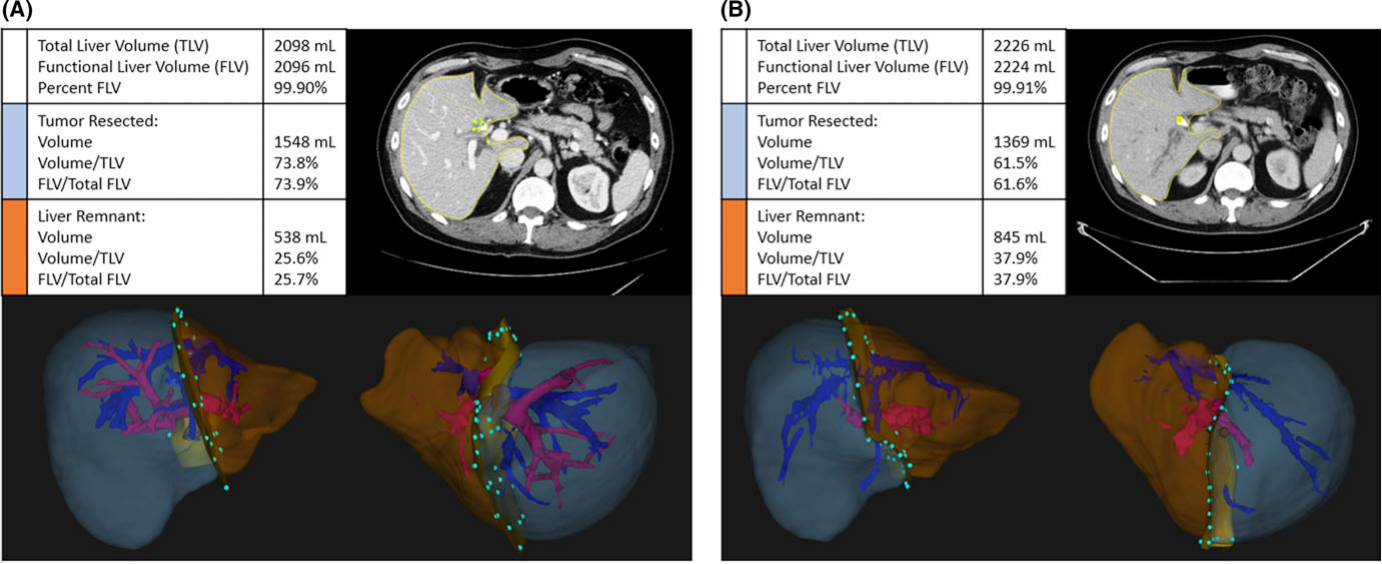
Volumetric analysis before A, and after B, portal vein embolization (PVE)

Patients requiring right hepatectomy or extended resection for hilar cholangiocarcinoma, in the absence of left liver atrophy, are at increased risk for postoperative hepatic insufficiency, as a sequela of inadequate liver remnant. To determine the role of FLR augmentation with PVE and/or biliary drainage, it is our practice to calculate the size of the FLR using volumetric analysis.^16^ Semiautomated software (Scout ™) is used to outline the contour of the liver, intrahepatic vasculature, and tumor, which allows for a three‐dimensional calculation of functional liver that will remain after resection, as a percentage of total preoperative functional liver (Figure 2).^16^ Shoup et al^16^ reported that a FLR less than 25% triples the risk of postoperative hepatic insufficiency and is predictive of morbidity and length of hospitalization. In the same study, 90% of patients that underwent trisectionectomy but had a FLR of 25% or less experienced postoperative hepatic insufficiency, compared to none of those with a FLR greater than 25%.^16^ Based on our experience at MKSCC, as well as the pioneering work on the implication of FLR volume by Vauthey, Makuuchi, and others, preoperative volumetric evaluation of the FLR is a key component of our preoperative assessment for patients that will require major hepatectomy.^16^, ^17^, ^18^, ^19^ Patients with a small FLR are referred for portal venous embolization and biliary drainage prior to curative intent resection (see below).^16^, ^20^

### 2.3 Optimization of the future liver remnant: Portal vein embolization and biliary drainage

Portal vein embolization and preoperative biliary drainage are two commonly used techniques to augment the FLR in preparation of major hepatectomy. From our observations of ipsilateral portal vein occlusion by the tumor with resultant contralateral hypertrophy, preoperative PVE is now carried out to recapitulate this phenomenon in preparation for surgery. First described by Makuuchi et al,^20^ preoperative PVE is considered when the FLR is anticipated to be small, and concerns for inadequate remnant function exist. In our experience, we find PVE to be especially important when the FLR is anticipated to be less than 25% of the total liver volume in healthy liver, less than 30% in the setting of chemotherapy‐induced liver toxicity, or less than 40% in compromised liver as a result of underlying cirrhosis.^21^, ^22^ Preoperative PVE is also particularly beneficial for patients with underlying hepatic insufficiency from cholestatic jaundice, cirrhosis, or steatosis (non‐alcoholic steatohepatitis [NASH] or preoperative chemotoxicity).^23^ Patients with hilar cholangiocarcinoma most commonly present to our institution with significant cholestasis and/or require neoadjuvant chemotherapy for locally advanced disease, and it is therefore our preference to carry out PVE prior to resection in patients with a predicted FLR less than 40%. PVE can be carried out efficiently with a minor complication profile that may include fever, pain, nausea, or bile leak, which occur in up to 12% of patients.^23^ Major complications of PVE, such as major hemorrhage or thrombus propagation into the main or FLR portal vein, are exceedingly rare (<1%).^23^ After PVE, contralateral lobar hypertrophy of the FLR ensues over the following 2 to 3 weeks, and augmentation of function is suggested by the kinetic growth rate of the FLR. Shindoh et al^24^ reported on 107 patients that underwent resection for colorectal liver metastases, in which a post‐PVE kinetic growth rate of the FLR exceeding 2% per week correlated with reduced hepatic insufficiency rates (0% vs 21.6%) and short‐term liver‐specific mortality (0% vs 8.1%). In fact, the kinetic growth rate was more predictive of such outcome measures than FLR volume or the overall degree of remnant hypertrophy.^24^ A contemporary analysis from MSKCC included 153 patients that underwent major hepatectomy (>3 segments) for primary and secondary malignancies after PVE.^25^ Based on this analysis, the degree of hypertrophy and growth rate were predictive of postoperative complications and liver failure.^25^ In fact, no patient with a kinetic growth rate exceeding 2.66% per week experienced postoperative liver failure.^25^ Patients with growth rates that fail to reach this threshold, or who do not achieve the expected degree of hypertrophy, are still considered for surgery, but an increased risk for postoperative morbidity should be anticipated.

It has long been suggested that preoperative obstructive jaundice is a risk factor for postoperative mortality in patients undergoing resection.^26^, ^27^, ^28^ Selective preoperative biliary drainage is therefore used to improve the safety of major hepatectomy for hilar cholangiocarcinoma. The most obvious indication for preoperative biliary drainage is decompression of the biliary tree in the setting of obstructive cholangitis. Additionally, biliary drainage can relieve symptoms such as pruritus in patients who will experience a delay in surgery. Even in patients that will undergo up‐front surgery, biliary drainage of a cholestatic liver facilitates normalization of hepatic function, which, if not attained, will yield an operative risk that is prohibitively high. Similarly, patients that will be managed with neoadjuvant chemotherapy should undergo biliary drainage, also to alleviate cholestasis and restore hepatic function in order to tolerate systemic therapy.

**Figure 3:**
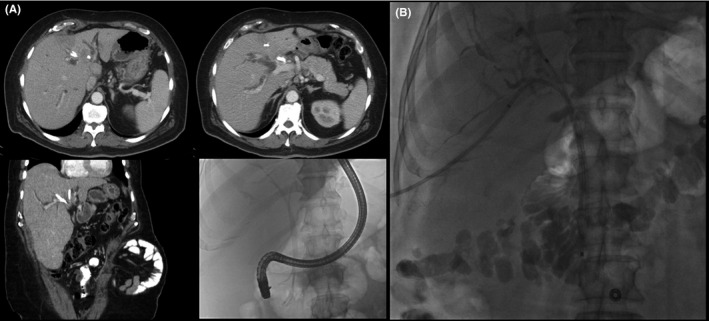
A, Computed tomography and fluoroscopic images showing inappropriate placement of three endoscopic stents in the atrophic left liver (planned resection), without adequate drainage of the future liver remnant (FLR). B, Fluoroscopic image showing super‐selective percutaneous placement of transhepatic catheters into the right anterior and posterior divisions of the FLR

Prior to discussing patient selection for preoperative biliary drainage, it is necessary to review two nuances related to such intervention. First, the FLR should be prioritized for drainage, rather than the biliary tree within the proposed parenchymal resection. In doing so, decompression of the remnant will aid in restoring metabolic and synthetic liver function within the remnant, as well as minimize the potential for atrophy as a sequela of chronic biliary obstruction. Additional drainage of the ipsilateral biliary tree may also be necessary to alleviate significant cholestatic jaundice that may preclude safe resection or administration of chemotherapy. Second, we prefer percutaneous transhepatic biliary drainage rather than endoscopic decompression. Transhepatic drainage for hilar cholangiocarcinoma allows for placement of an internal‐external drain or primary wall stent such that the distal end terminates above the ampulla. In doing so, catheter patency is improved and the risk of ascending contamination with intestinal flora is minimized.^29^ Patency is particularly important for patients that require neoadjuvant therapy. Additionally, super‐selective placement of biliary drains into segmental bile ducts can be achieved percutaneously with the assistance of CT and fluoroscopy, which is much more challenging endoscopically (Figure 3). In our experience, endoscopic drainage more often leads to errant placement stents, leading to cholangitis and requiring further procedures to optimize drainage.^30^ If transhepatic drainage is not possible, endoscopic stenting can be carried out, but it should be noted that this technique may result in recurrent episodes of cholangitis, not only delaying definitive resection but also increasing the morbidity of the operation and postoperative length of hospitalization.^29^ Some authors have reported an increase in tumor seeding with percutaneous drainage, but we have not had this experience.^31^

The goal of preoperative biliary drainage was to improve the function of the FLR to reduce the risk of postoperative liver failure; however, the literature is inconclusive with regard to the optimal duration of drainage or the serum bilirubin level that should be achieved prior to resection. Although it has been suggested that operation should be delayed until the bilirubin level decreases to less than 3.0 mg/dL, this is not based on any rigorous assessment of hepatic function, many aspects of which are not measurable with standard clinical tests.^32^ Several studies have presented strong arguments for a selective approach to preoperative biliary drainage, depending on the size of the FLR.^33^, ^34^ The purpose of the 2009 analysis by Kennedy et al^33^ was to determine the impact of the FLR volume and preoperative biliary drainage on postoperative outcomes, specifically postoperative hepatic insufficiency and mortality. Of the 60 patients included in the analysis, only 63% underwent preoperative biliary drainage of the FLR, which included transhepatic drainage in 67% and endoscopic stenting in 29%.^33^ In the group with a FLR exceeding 30%, no patients experienced postoperative hepatic insufficiency; however, the mortality rate was 9% in this population that received biliary drainage, compared to 0% for those that did not.^33^ The opposite was observed in patients with a FLR less than 30%; postoperative hepatic insufficiency and mortality rates were 33% each for those in which biliary drainage was omitted, compared to 0% hepatic insufficiency and 0% mortality for patients with small future remnants that underwent FLR drainage prior to surgery.^33^ Although a small study, it showed that preoperative biliary drainage may improve outcomes for patients with small FLR, but may also be detrimental to those with remnants of 30% or larger.^33^ In a subsequent analysis of hilar cholangiocarcinoma patients at MSKCC and the Academic Medical Center in Amsterdam, postoperative mortality was calculated to be 14%, predicted by increasing age, preoperative cholangitis, FLR <30%, need for portal vein reconstruction, and incomplete drainage of the biliary tree in patients with FLR less than 50%.^34^ There was a clear correlation with improved outcomes after preoperative biliary drainage in patients with a FLR <30%; however, Wiggers et al^34^ also concluded preoperative biliary drainage to be detrimental to patients with remnants >50% in which drained patients experienced higher rates of cholangitis (20% vs 8%) and mortality (12% vs 0%). Based on these studies, it is our practice to selectively drain the remnant of patients with an estimated FLR smaller than a minimum of 40% while taking into account the patient's risk for underlying hepatic dysfunction.

### 2.4 Role of neoadjuvant chemotherapy for hilar cholangiocarcinoma

Patients with resectable hilar cholangiocarcinoma should be recommended for up‐front surgery, rather than treating them with neoadjuvant therapy. This approach is supported by retrospective data that showed an 11‐month median survival advantage for patients that were resected without neoadjuvant therapy, which can delay resection by nearly 7 months.^35^ Unfortunately, nearly 65% of patients do not have resectable disease at presentation.^7^ Nearly 15% of patients with hilar cholangiocarcinoma will be deemed unresectable at presentation as a result of evidence of locally advanced disease on cross‐sectional imaging.^7^ Additionally, of patients explored with curative intent, nearly 20% will have unresectable disease as a result of local invasion.^7^ With surgical resection representing the only means to a cure, neoadjuvant therapy may be used selectively in patients with locally advanced hilar cholangiocarcinoma in an attempt to downstage the disease and convert to resectable status.^36^ Without randomized data to support the use of neoadjuvant therapy, we are left with retrospective and small prospective pilot studies to drive the decision to treat patients with chemotherapy and/or radiation therapy first, followed by surgery.^37^, ^38^ Such data do suggest an improved R0 resection rate when locally advanced hilar cholangiocarcinoma is treated with neoadjuvant therapy.^36^, ^37^, ^38^

Patients with locally advanced hilar cholangiocarcinoma that are managed with neoadjuvant therapy should undergo pretreatment biliary drainage, ideally by percutaneous transhepatic technique.^29^ As stated above, transhepatic stenting or drainage preserves ampullary integrity, in order to avoid contamination of the biliary tree that occurs with endoscopic stenting.^29^ Additionally, the chemotherapy of choice that is used in the preoperative setting for hilar cholangiocarcinoma is an extrapolation of prospective randomized data pertaining to locally advanced and metastatic biliary tract cancers.^11^ The ABC‐02 trial, which included patients with cholangiocarcinoma, gallbladder cancer, and ampullary cancer, demonstrated an improvement in progression‐free survival and tumor control, as well as a survival advantage of 3.5 months for patients treated with a combination of gemcitabine and cisplatin, compared to gemcitabine monotherapy.^11^

## 3 SURGICAL APPROACH AND INTRAOPERATIVE CONSIDERATIONS

The conduct of the operation should begin with selective diagnostic laparoscopy (see below) followed by partial hepatectomy with en bloc resection of the extrahepatic bile duct, portal lymphadenectomy, and Roux‐en‐Y hepaticojejunostomy. It should be noted that the right hepatic duct is short with significant anatomical variability, adding to the complexity of left‐sided resections, whereas the left hepatic duct is several centimeters in length and follows a more predictable course, making right hepatectomies more straightforward. Additionally, biliary drainage of the caudate lobe typically drains into the left biliary tree or biliary confluence and lies in close proximity to the right hepatic artery. Therefore, the caudate lobe should be resected en bloc with all centrally located and left‐sided tumors, and selectively for right‐sided tumors when the caudate duct drains into the right biliary system. Following these criteria for caudate resection may minimize the risk of biliary leak from an uncontrolled caudate duct as well as reduce the risk for local recurrence from a positive margin.

### 3.1 Diagnostic laparoscopy, vascular control, and parenchymal transection

Surgery for hilar cholangiocarcinoma starts with selective diagnostic laparoscopy for Blumgart T2 and T3 lesions, or if there is concern for advanced disease on preoperative imaging.^15^ In such cases, laparoscopy detects occult peritoneal metastases in over 10% of patients, thereby sparing these patients a non‐therapeutic laparotomy.^39^ The subsequent incision used for the laparotomy should be appropriate for the planned resection and body habitus of the patient, in order to provide adequate exposure and access to the small intestine. Intraoperative ultrasound is used to confirm anatomical relationships and biliary tumor extent suggested on preoperative imaging and to rule out intrahepatic metastases. Hilar dissection is carried out to clear all lymphatic tissues and to expose the portal structures. The distal bile duct is divided early on in the dissection to allow better access to the vascular structures. Frozen section of the distal bile duct is carried out to ensure the absence of tumor involvement. The ipsilateral hepatic artery and portal vein are ligated and divided extrahepatically, leaving division of bile duct to be done sharply during parenchymal transection. The ipsilateral hepatic vein is also preferentially divided as it enters the inferior vena cava, after inflow control but prior to hepatic parenchymal transection.

Parenchymal transection is carried out with intermittent Pringle maneuver, under low central venous pressure, which is not only safe but also reduces intraoperative blood loss.^40^ The technique for transection combines crush clamping, division of crossing vascular structures and ducts between clips or ties, as well as the stapler.

### 3.2 Vascular resection

Complete extirpation of the tumor may require en bloc portal vein resection and reconstruction, which is particularly true for advanced tumors.^41^ In a multi‐institutional analysis, median survival after an R0 resection was 27.7 months, compared to 14.1 months after an R1 resection.^41^ Portal vein resection may be necessary to achieve negative margins and optimize long‐term survival.^42^ Although some experienced centers have reported portal vein resection without an increase in perioperative mortality, other centers that practice the “no touch technique” have seen postoperative mortality rates as high as 10%‐13%, depending on the volume of liver resected.^41^, ^43^ Portal vein resection may result in improved oncological outcomes for selected patients; however, the potential short‐term risk to the patient must be appreciated.^42^, ^43^

Although portal vein resection has been deemed reasonable for highly selected patients at experienced centers, arterial resection is championed by few. The largest experience of this technically demanding maneuver comes from Japan, where Nagino and colleagues have suggested that arterial resection, oftentimes with venous resection, is necessary to completely extirpate the tumor.^44^ In their report of 50 patients undergoing simultaneous arterial and venous resection, median operative time approached 13 hours, median blood loss exceeded 2.5 liters, and patients stayed in the hospital a median of 32 days postoperatively.^44^ Re‐exploration was necessary in 10% of their series with an overall morbidity rate of 54%, including 7% with postoperative hepatic insufficiency.^44^ Despite the technical demand of these operations, only one patient (2%) died postoperatively.^44^ Furthermore, from an oncological perspective, 5‐year survival was 50% better for patients requiring resection of both the hepatic artery and portal vein, as compared to no vascular resection at all.^44^ Regardless of their success, arterial involvement to the FLR is considered unresectable disease at MSKCC and at most other institutions, and these patients are therefore referred for alternative therapies.

### 3.3 Intraoperative assessment of the bile duct margin

Intraoperative assessment of the bile duct margin should be carried out, but with caution and an understanding of the limitations of frozen section. A positive bile duct margin is more likely a marker of disease biology than an inadequate resection. Additionally, the bile duct resection margin represents only one aspect; the radial margin in the porta hepatis and the hepatic parenchymal transection line are equally important pathological parameters but are not typically assessed. Retrospective analysis of 101 patients treated at MSKCC from 1992 to 2005 divided patients into three groups based on margin status: (i) wide margin (bile duct and specimen margin were both negative); (ii) narrow margin (bile duct margin was negative, but specimen margin was positive); and (iii) positive margin (bile duct and specimen margin were positive).^45^ It should be noted that 9% of patients that had an intraoperative negative margin actually had a positive margin on final pathology.^45^ After final pathological analysis, the disease‐specific survival was 54 months for patients resected with a wide margin compared to 38 months after a narrow margin and 32 months after a positive margin (*P* = .01).^45^ Furthermore, when negative margins were achieved after an initially positive margin, survival was similar to that of patients with a definitively positive margin.^45^

A contemporary analysis by Tsukahara et al addresses intraoperative bile duct margin assessment but is somewhat contradictory to the analysis from MSKCC.^46^ The retrospective analysis from Japan reports decreased recurrence and improved disease‐specific survival for patients that underwent reresection for focal in situ disease to yield a final negative margin.^46^ It should be noted, however, that the intraoperative diagnosis of in situ disease was falsely positive in 11% of patients, subjecting these patients to an increased risk of morbidity associated with more extensive resection (morbidity not addressed in the publication).^46^ Additionally, 42% of patients that underwent additional resection still had a focus of disease on the final margin analysis.^46^

Although the Japanese group recommends additional resection for in situ disease that is identified intraoperatively, we do not universally share this opinion. In our experience, additional resection does not provide a survival advantage and, therefore, chasing margins is not recommended in most cases. Rather, the initial resection should be conducted with the widest margin that is technically achievable, while taking into account the morbidity and risk for postoperative hepatic insufficiency associated with excessive parenchymal resection.^45^

### 3.4 Reconstruction and operative drain placement

Reconstruction of biliary‐enteric drainage is typically carried out with a retrocolic Roux‐en‐Y hepaticojejunostomy. The end‐to‐side duct‐to‐mucosa anastomosis is typically created with interrupted 4‐0 dissolvable monofilament suture (ie, polydioxanone). To minimize bile reflux, a 60‐cm Roux limb is used to separate the biliary and gastropancreatic contents, thereby reducing the risk of bile reflux. After inspection of the anastomosis, a single 19 Fr channel drain is positioned anterior to the biliary anastomosis and along the raw hepatic parenchymal surface, which is subsequently removed postoperatively when there is no evidence of biliary leak.

## 4 POSTOPERATIVE CONSIDERATIONS AND ADJUVANT THERAPY

### 4.1 Postoperative hepatic insufficiency

Parenchymal preservation in the modern era of hepatic resection has likely contributed to observed reduction in postoperative morbidity, hepatic insufficiency, and liver‐related death.^3^ Unfortunately, resection of hilar cholangiocarcinoma requires major hepatectomy in nearly all cases. It is well established that the risk for postoperative hepatic insufficiency is linked with the amount of functional parenchyma resected or, in other words, the size of the liver remnant.^3^ Knowing this, a left hepatectomy with en bloc caudate resection is generally a safer procedure, as the volume of left liver is lower than the right, and the volume of the right liver remnant is usually adequate. For patients requiring right hepatectomy or an extended resection, preoperative optimization of the FLR with PVE and/or biliary drainage can further reduce the risk of hepatic insufficiency. Postoperatively, recognition of synthetic and metabolic dysfunction is imperative such that best supportive care can be provided. A patient that meets criteria for the 50:50 rule, which includes protrombin time <50% and bilirubin >50 μmol/L between postoperative days 3 to 8, is at an increased risk for postoperative mortality.^47^ Additionally, based on our own institutional data, rising creatinine and failure of serum phosphorus to decrease by 20% on postoperative day 1 are also predictive of postoperative hepatic insufficiency.^48^ Although recovery of hepatic function is a process that evolves naturally, supportive care with early recognition and prompt treatment of additional complications is essential to rescue patients from subsequent mortality.

### 4.2 Adjuvant therapy

Until recently, there were no randomized data to support the use of adjuvant therapy for hilar cholangiocarcinoma, even in high‐risk patients. However, at MSKCC, high‐risk patients (positive margins and/or lymph node metastases) have generally been recommended to consider gemcitabine‐based adjuvant chemotherapy and/or radiation.^12^ The use of gemcitabine and cisplatin in the adjuvant setting is an extrapolation from the randomized data for metastatic biliary cancers.^11^ More recently, Primrose et al presented randomized data supporting adjuvant capecitabine for biliary tract cancers, including hilar cholangiocarcinoma (ASCO Abstract 4006, 2017). In the this study (BILCAP trial), patients with biliary tract cancers were randomized to observation or adjuvant capecitabine, which indicated a 15‐month median survival advantage for those that received drug (51 months vs 36 months). Recurrence‐free survival also favored the treatment arm, which was improved to 25 months, compared to 18 months for the observation arm. Given these randomized data, capecitabine in the adjuvant setting has become the standard of care for all patients. Additionally, for patients with residual disease (positive margins) or for those at high risk of recurrence (positive nodes), adjuvant radiation therapy may also be considered, although there are no randomized data to support this strategy.^12^, ^49^

## 5 CONCLUSIONS

Although the majority of patients with hilar cholangiocarcinoma present with locally advanced or metastatic disease, aggressive multidisciplinary management can result in long‐term survival for those with resectable disease. A critical assessment of the patient's preoperative imaging is necessary to determine resectability. Augmentation of the FLR with PVE and biliary drainage should be considered in the treatment strategy, especially for patients undergoing extended resections resulting in a small FLR and/or those with underlying hepatic insufficiency from processes such as cholestasis. Definitive resection, combined with adjuvant therapy to reduce the risk of recurrence, does offer patients with hilar cholangiocarcinoma an opportunity for long‐term survival and should be the standard approach for selected patients. Liver transplantation has a limited but important role, primarily in patients with underlying liver disease.

## DISCLOSURE

Conflicts of Interest: Authors declare no conflicts of interest for this article.
